# Higher exposure to 1,3-butadiene is associated with more severe hearing loss

**DOI:** 10.1038/s41598-024-63757-7

**Published:** 2024-06-05

**Authors:** Sang-Yoon Han, Sang-Yeon Lee, Myung-Whan Suh, Jun Ho Lee, Moo Kyun Park

**Affiliations:** 1 Department of Otolaryngology-Head and Neck Surgery, College of Medicine, Hanyang University, Seoul, Republic of Korea; 2 Department of Otorhinolaryngology-Head and Neck Surgery, Seoul National University Hospital, 101 Daehak-Ro, Jongno-gu, Seoul, Republic of Korea; 3 Medical Research Center, Sensory Organ Research Institute, Seoul National University, Seoul, Republic of Korea

**Keywords:** Sensorineural hearing loss, High-frequency hearing loss, Volatile organic compounds, Environmental pollutants, Noise, Diseases, Neurological disorders, Environmental sciences, Environmental chemistry, Disease prevention, Occupational health, Public health

## Abstract

While volatile organic compounds (VOCs) impair various organs, their influence on hearing loss (HL) has not been extensively researched. We aimed to identify the association between VOCs and HL or high-frequency hearing loss (HFHL). We extracted data on age, sex, pure tone audiometry, hypertension, occupational noise exposure, and creatinine-corrected urine VOC metabolite concentrations from the eighth Korea National Health and Nutrition Survey. Among the VOC metabolites, N-acetyl-S-(benzyl)-L-cysteine (BMA, *P* = 0.004), N-acetyl-S-(phenyl)-l-cysteine (SPMA, *P* = 0.027), and N-acetyl-S-(3,4-dihydroxybutyl)-l-cysteine (DHBMA, *P* < 0.001) showed associations with HL. Additionally, HFHL exhibited significant associations with BMA (*P* = 0.005), 3- and 4-methylhippuric acid (3, 4 MHA, *P* = 0.049), mandelic acid (MA,* P* = 0.015), SPMA (*P* < 0.001), N-acetyl-S-(3-hydroxypropyl)-l-cysteine (3-HPMA, *P* < 0.001), and DHBMA (*P* < 0.001). After controlling other factors, DHBMA were associated with HL (*P* = 0.021) and HFHL (*P* = 0.014) and exhibited a linear association with the mean hearing level (β = 0.054, *P* = 0.024) and high-frequency hearing level (β = 0.045, *P* = 0.037). Since 1,3-butadiene may act as an ototoxic material, early screening for workers exposed to 1,3-butadiene and reducing exposure to 1,3-butadiene in everyday life may be helpful to prevent further HL.

## Introduction

Environmental pollutants have negative effects on an individual’s physical health^1,2^. They can cause harm to the organ they come into direct contact with, as well as systemic damage, including cardiovascular disease, liver damage, renal impairment, and impairments to the central or peripheral nervous system^1,2^. Volatile organic compounds (VOCs), such as acrolein, acrylamide, 1,3 butadiene, toluene, and benzene, are environmental pollutants produced as byproducts of industrial processes^1,3^. It originates from natural sources and industrial sources during the synthesis of materials or manufacturing products such as synthetic rubber, resin, pesticides, and paints^3^ VOCs also negatively impact health, potentially causing headaches, nausea, vomiting, dizziness, atopic dermatitis, and otitis media, and are associated with an increased risk of cancer^3–5^. Furthermore, VOCs can cause neuronal damage, which may result in dysfunction of the central and peripheral nervous systems^3,4^.

Previous studies have established that sensorineural hearing loss (HL) is associated with a variety of environmental factors. Noise exposure is a well-known factor contributing to HL^4,6^. Moreover, research has indicated that exposure to certain heavy metals, such as cadmium and lead, is significantly associated with HL^7–9^. Long and Tang also found that organochlorine pesticides are linked to hearing impairment^10^. Additionally, the relationship between HL and certain types of air pollutants has been explored. Some studies have shown that environmental pollutants like PM_10_, NO_2_, CO, and SO_2_ can adversely affect hearing^11,12^.

However, there have been few studies on the relationship between VOCs and hearing thresholds A previous study demonstrated that the co-exposure of VOCs and/or heavy metals with noise exposure may exacerbate the risk of noise-induced hearing loss in occupational workers^13^. Additionally, only a handful of studies have described an association between higher levels of VOCs, in the absence of noise exposure, and high-frequency hearing loss (HFHL)^14,15^. Furthermore, one of those studies did not consider middle ear status, which can be affected by VOCs^15^. Given that VOCs can induce nerve damage, including damage to central and peripheral nerves, as well as DNA damage to various types of cells^3,4^, they may have detrimental effects on the auditory system. Additionally, VOCs can be easily exposed to in both indoor and outdoor environments, they can have effects on the hearing of the general population^3^. In this study, we aimed to evaluate the association between VOCs and HL in the general population. Additionally, we assessed the same potential association with HFHL because the basal hair cells are more susceptible to environmental ototoxic factors, such as reactive oxygen species, noise, and ototoxic drugs^16–18^.

## Materials and methods

### Database and subject inclusion

We extracted all data and subjects from the eighth Korean National Health and Nutrition Examination Survey (KNHANES) conducted in 2021. The KNHANES was conducted to evaluate the health and nutritional status of the general population using a two-stratified random-sampling method. The database provided information on subjects’ age, sex, household income (quintile), history of occupational noise exposure, results of pure tone audiometry and tympanometry, as well as concentrations of VOC metabolites in urinary analysis. Occupational noise exposure was evaluated based on the survey questions. Pure tone audiometry was carried out using an AD629 audiometer (Interacoustics, Assens, Denmark) inside a 20 cm double-wall soundproof booth, while impedance was measured with a Titan IMP440 screener (Interacoustics, Assens, Denmark). The VOC metabolites included in this survey were N-acetyl-S-(benzyl)-L-cysteine (BMA), 2-methylhippuric acid (2-MHA), 3- and 4-methylhippuric acid (3, 4 MHA), phenylglyoxylic acid (PGA), mandelic acid (MA), N-acetyl-S-(phenyl)-l-cysteine (SPMA), N-acetyl-S-(3-hydroxypropyl)-l-cysteine (3-HPMA), N-acetyl-S-(n-propyl)-l-cysteine (BPMA), and N-acetyl-S-(3,4-dihydroxybutyl)-l-cysteine (DHBMA). These VOCs were analyzed using liquid chromatography-mass spectrometry with the Nexera XR LC-20AD System (Shimadzu, Kyoto, Japan) and the Triple Quad API 5500 (Sciex, Framingham, USA). Before measuring the VOC metabolites, the liquid chromatography-mass spectrometry system was calibrated using an internal standard, achieving a mean accuracy ranging from 91.6% to 107.1%, depending on the specific VOC metabolite. The concentrations of VOC metabolites were normalized to creatinine levels. The eighth KNHANES received approval from the Institutional Review Board of Korea Disease Control and Prevention Agency (IRB No. 2018-01-03-5C-A). All participants were provided with an explanation of the study and gave their informed consent prior to participation. The KNHANES was conducted following regulations and guidelines provided by the Korea Disease Control and Prevention Agency, and our study was performed in accordance with the STROBE statement.

### Data selected for analysis

We extracted data on age, sex, pure tone audiometry, and tympanic membrane status, which was evaluated using tympanometry, from the database. Since VOCs have a significant effect on cardiovascular disease, we also included data on hypertension^19–21^. We also extracted data on occupational noise exposure history because workers with a high risk of VOC exposure may also be exposed to occupational noise^6,22^, which could act as a confounding factor.

### Classification of hearing loss groups

We calculated hearing thresholds by averaging the results of pure tone audiometry at 0.5 kHz, 1 kHz, 2 kHz, and 4 kHz. We defined high-frequency (HF) hearing thresholds as the average of pure tone audiometry results at 2 kHz, 4 kHz, and 8 kHz. Subsequently, subjects were categorized into three groups according to their hearing levels in the worse ear following the WHO classification^23,24^: the normal hearing group, which includes individuals with hearing thresholds of 25 dB or less; the mild HL group, consisting of individuals with hearing thresholds greater than 25 dB but not exceeding 40 dB; and the moderate to severe HL group, comprising individuals with hearing thresholds above 40 dB. In addition, we applied the same classification criteria (normal HF hearing group, mild HFHL group, moderate to severe HFHL group) to categorize individuals based on their HF hearing thresholds.

### Statistical analysis

To evaluate the associations between each group and VOC metabolites, analysis of variance (ANOVA) was conducted. For VOC metabolites that showed a significant association, a multivariate analysis of covariance (MANCOVA) was performed to control for other associated factors. The Bonferroni test was applied for post-hoc analyses following MANCOVA. The linear regression analysis and binary logistic regression analysis were conducted to evaluate the association of each VOC with HL or HFHL after adjusting for other variables. Additionally, quantile g-computation was performed using VOCs with a *P*-value less than 0.1 in age-and sex-adjusted analysis to reduce dimensionality and address collinearity issues raised by single pollutant models. A *P*-value of less than 0.05 was considered to indicate a statistically significant difference. According to the central limit theorem, normality tests were not conducted, and normal statistical analyses were performed for sample sizes equal to or exceeding 30 individuals^25^. All statistical analyses were performed using SPSS version 25.0 (IBM Corp., Armonk, NY, USA) or R, version 4.4.0 (R Project for Statistical Computing).

## Results

### Inclusion process of subjects and the demographic factors, hypertension, and occupational noise exposure history of each group

**Figure 1:**
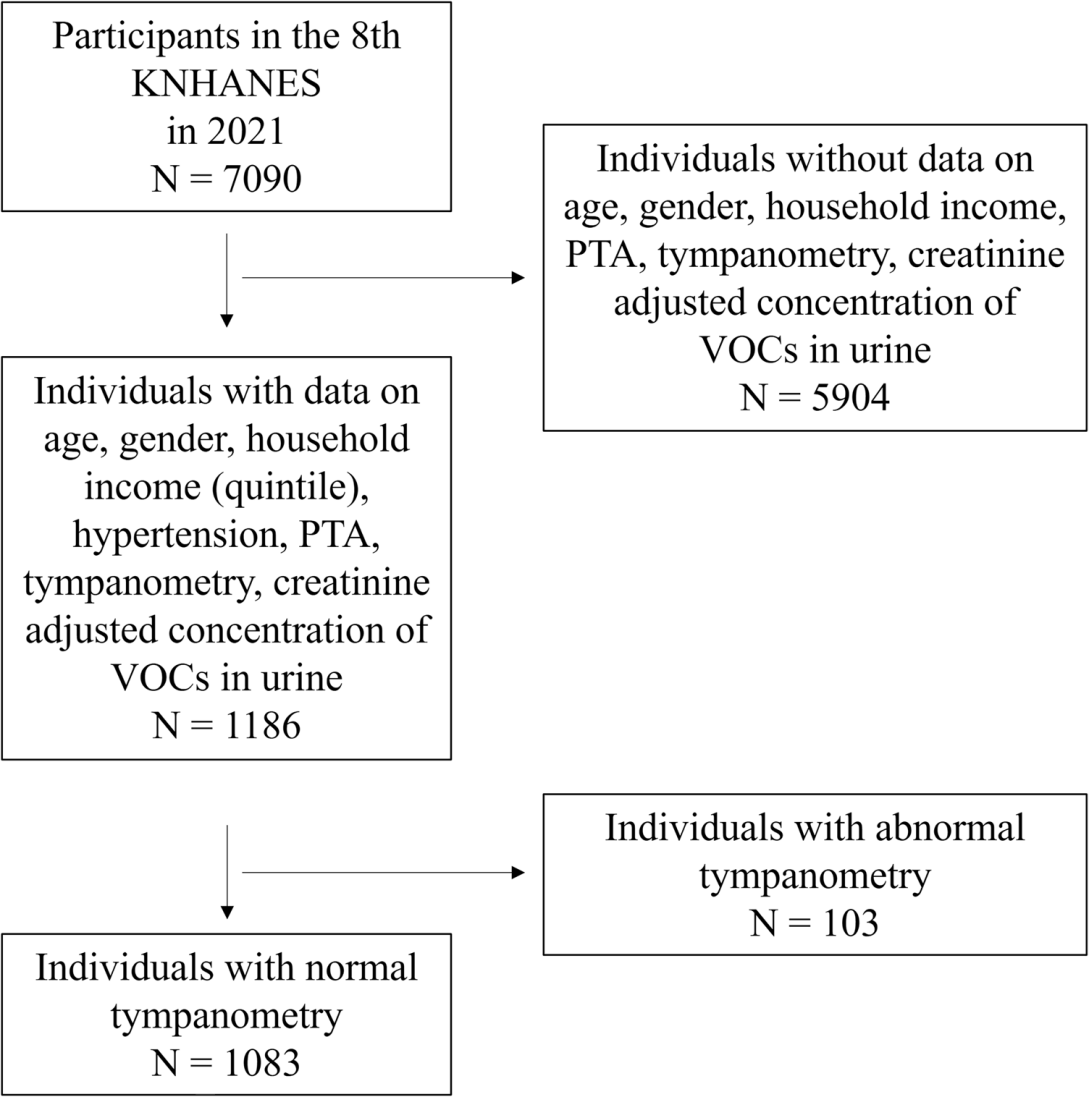
Inclusion process of subjects from database KNHANES, Korea National Health and Nutrition Examination Survey; *N* number, *PTA* pure tone audiometry; *VOCs* volatile organic compounds.

The total number of participants was 7090 in the eighth KNHANES in 2021. Of these, 1186 participants with data on household income, pure tone audiometry, tympanometry, and urinary concentrations of VOC metabolites were selected. Subsequently, 103 individuals with abnormal tympanometry results were excluded. Ultimately, 1083 subjects were included in this study (Fig. 1).

All subjects were aged 40 or older, as the hearing tests in the eighth KNHANES were only administered to individuals within this age range. The mean age of the subjects was significantly different between the HL group (*P* < 0.001) and the HFHL group (*P* < 0.001) (Table 1). Their household income was also different among the HL groups (*P* < 0.001) and the HFHL groups (*P* < 0.001) (Table 1). Furthermore, significant differences were observed in sex distribution, hypertension prevalence, and history of occupational noise exposure between the HL groups (*P* < 0.001 for sex; *P* < 0.001 for hypertension; *P* < 0.001 for occupational noise exposure) and the HFHL groups (*P* < 0.001 for sex; *P* < 0.001 for hypertension; *P* = 0.006 for occupational noise exposure) (Table 1).

**Table 1:** Demographic factors, hypertension, and occupational noise exposure of each group.

Variable	Hearing loss				High-frequency hearing loss			
	None (N = 688)	Mild (N = 255)	Moderate to severe (N = 140)	*P*-value	None (N = 439)	Mild (N = 246)	Moderate to severe (N = 398)	*P*-value
Age (years)	55.25 ± 10.46	66.11 ± 9.32	70.49 ± 9.52	< 0.001	50.92 ± 8.36	61.97 ± 9.02	68.18 ± 9.51	** < 0.001**
Sex (M : F)	266 : 422	130 : 125	81 : 59	< 0.001	149 : 290	91 : 155	237 : 161	**< 0.001**
Household income (quintile)	3.47 ± 1.30	2.76 ± 1.39	2.28 ± 1.19	< 0.001	3.74 ± 1.17	3.07 ± 1.38	2.56 ± 1.32	**< 0.001**
Hypertension	22.82%	45.88%	50.71%	< 0.001	14.58%	36.59%	47.99%	**< 0.001**
Occupational noise exposure	14.10%	20.00%	23.57%	0.006	11.62%	16.67%	22.36%	**< 0.001**

*M* male, *F* female.

Significants values are in bold.

### Univariable analysis and crude analysis of the association between hearing thresholds and each VOC metabolite

In the univariable analysis, the HL groups showed significantly different creatinine-corrected concentrations of BMA (*P* = 0.004), SPMA (*P* = 0.027), and DHBMA (*P* < 0.001) (Table 2). In addition, the HFHL groups exhibited significant differences in the concentrations of BMA (*P* = 0.005), 3, 4 MHA (*P* = 0.049), MA (*P* = 0.015), SPMA (*P* < 0.001), 3-HPMA (P < 0.001), and DHBMA (*P* < 0.001) (Table 2). Other VOCs did not show significant differences between the HL and HFHL groups (Table 2). Since the groups classified with HL or HFHL differed in age and sex, crude analyses were conducted for significant variables from the univariable study, controlling for age and sex. After adjusting for age and sex, only the creatinine-corrected concentration of DHBMA remained significantly different in the HL groups (*P* = 0.017) and the HFHL groups (*P* = 0.009). No other VOCs showed any differences among the HL or HFHL groups (Table 3).

**Table 2:** Mean values of each volatile organic compound and its association with hearing loss or high-frequency hearing loss.

VOCs	Hearing loss				High-frequency hearing loss			
	None (N = 688)	Mild (N = 255)	Moderate to severe (N = 140)	*P*-value	None (N = 439)	Mild (N = 246)	Moderate to severe (N = 398)	*P*-value
BMA (μg/g Cr)	8.68 ± 14.52	12.70 ± 28.05	12.48 ± 15.96	**0.004**	8.17 ± 15.11	9.91 ± 14.97	12.40 ± 23.83	**0.005**
2-MHA (μg/g Cr)	32.47 ± 164.17	40.65 ± 130.32	29.14 ± 34.60	0.684	33.16 ± 203.15	31.03 ± 43.70	36.68 ± 106.01	0.882
3, 4 MHA (μg/g Cr)	153.85 ± 284.12	200.81 ± 549.33	176.19 ± 225.22	0.195	135.61 ± 267.46	183.54 ± 317.99	193.56 ± 456.39	**0.049**
PGA (μg/g Cr)	293.75 ± 284.92	329.88 ± 336.75	312.12 ± 190.76	0.220	272.73 ± 303.93	316.17 ± 228.20	332.70 ± 300.60	**0.008**
MA (μg/g Cr)	229.52 ± 302.50	257.67 ± 262.64	290.79 ± 347.09	0.063	215.73 ± 324.23	243.48 ± 242.48	275.69 ± 302.92	**0.015**
SPMA (μg/g Cr)	1.0 0.55	1.11 ± 0.64	1.07 ± 0.51	**0.027**	0.93 ± 0.50	1.15 ± 0.62	1.08 ± 0.58	** < 0.001**
3-HPMA (μg/g Cr)	641.26 ± 826.94	726.61 ± 725.78	744.86 ± 599.69	0.170	551.92 ± 803.08	754.76 ± 641.07	760.77 ± 811.45	** < 0.001**
BPMA (μg/g Cr)	83.16 ± 110.50	87.14 ± 127.18	108.98 ± 146.19	0.067	80.63 ± 113.21	91.30 ± 111.65	92.55 ± 131.29	0.302
DHBMA (μg/g Cr)	259.31 ± 95.14	278.56 ± 117.45	310.84 ± 142.40	** < 0.001**	246.17 ± 86.03	280.89 ± 95.44	290.93 ± 132.27	** < 0.001**

*Cr* creatinine, BMA, N-acetyl-S-(benzyl)-L-cysteine; *2-MHA* 2-methylhippuric acid, 3,4 MHA, 3- and 4-methylhippuric acid; *PGA* phenylglyoxylic acid, *MA* mandelic acid, SPMA, N-acetyl-S-(phenyl)-l-cysteine, 3-HPMA, N-acetyl-S-(3-hydroxypropyl)-l-cysteine, BPMA, N-acetyl-S-(n-propyl)-l-cysteine, DHBMA, N-acetyl-S-(3,4-dihydroxybutyl)-l-cysteine.

Significants values are in bold.

**Table 3:** Age- and sex-adjusted mean values and standard error of each volatile organic compound and its association with hearing loss or high-frequency hearing loss.

VOCs (μg/g Cr)	Hearing loss			
	None (N = 688)	Mild (N = 255)	Moderate to severe (N = 140)	*P*-value
BMA	9.64 ± 0.75	11.34 ± 1.22	10.24 ± 1.69	0.525
SPMA (μg/g Cr)	1.06 ± 0.21	1.03 ± 0.34	0.94 ± 0.05	0.070
DHBMA (μg/g Cr)	265.90 ± 4.20	269.08 ± 6.78	295.76 ± 9.40	**0.017**

	High-frequency hearing loss			
	None (N = 439)	Mild (N = 246)	Moderate to severe (N = 398)	*P*-value
BMA (μg/g Cr)	10.23 1.07	9.06 ± 1.19	10.65 ± 1.11	0.573
3, 4 MHA (μg/g Cr)	128.45 ± 20.70	188.31 ± 23.03	198.52 ± 21.41	0.082
PGA (μg/g Cr)	289.91 ± 16.46	306.71 ± 18.31	319.60 ± 17.03	0.547
MA (μg/g Cr)	240.30 ± 17.19	233.97 ± 19.13	254.47 ± 17.79	0.722
SPMA (μg/g Cr)	1.04 ± 0.03	1.09 ± 0.03	1.00 ± 0.03	0.097
3-HPMA (μg/g Cr)	646.16 ± 44.38	738.23 ± 49.37	667.05 ± 45.92	0.339
DHBMA (μg/g Cr)	255.39 ± 5.95	273.86 ± 6.62	285.10 ± 6.16	**0.009**

*Cr* creatinine, *BMA* N-acetyl-S-(benzyl)-L-cysteine, *2-MHA* 2-methylhippuric acid, *3, 4 MHA* 3- and 4-methylhippuric acid, *PGA* phenylglyoxylic acid, *MA* mandelic acid, *SPMA* N-acetyl-S-(phenyl)-l-cysteine, *3-HPMA* N-acetyl-S-(3-hydroxypropyl)-l-cysteine, *BPMA* N-acetyl-S-(n-propyl)-l-cysteine, *DHBMA* N-acetyl-S-(3,4-dihydroxybutyl)-l-cysteine.

Significants values are in bold.

### Multivariable analysis of the VOC metabolites associated with hearing loss in the univariable analysis

**Figure 2:**
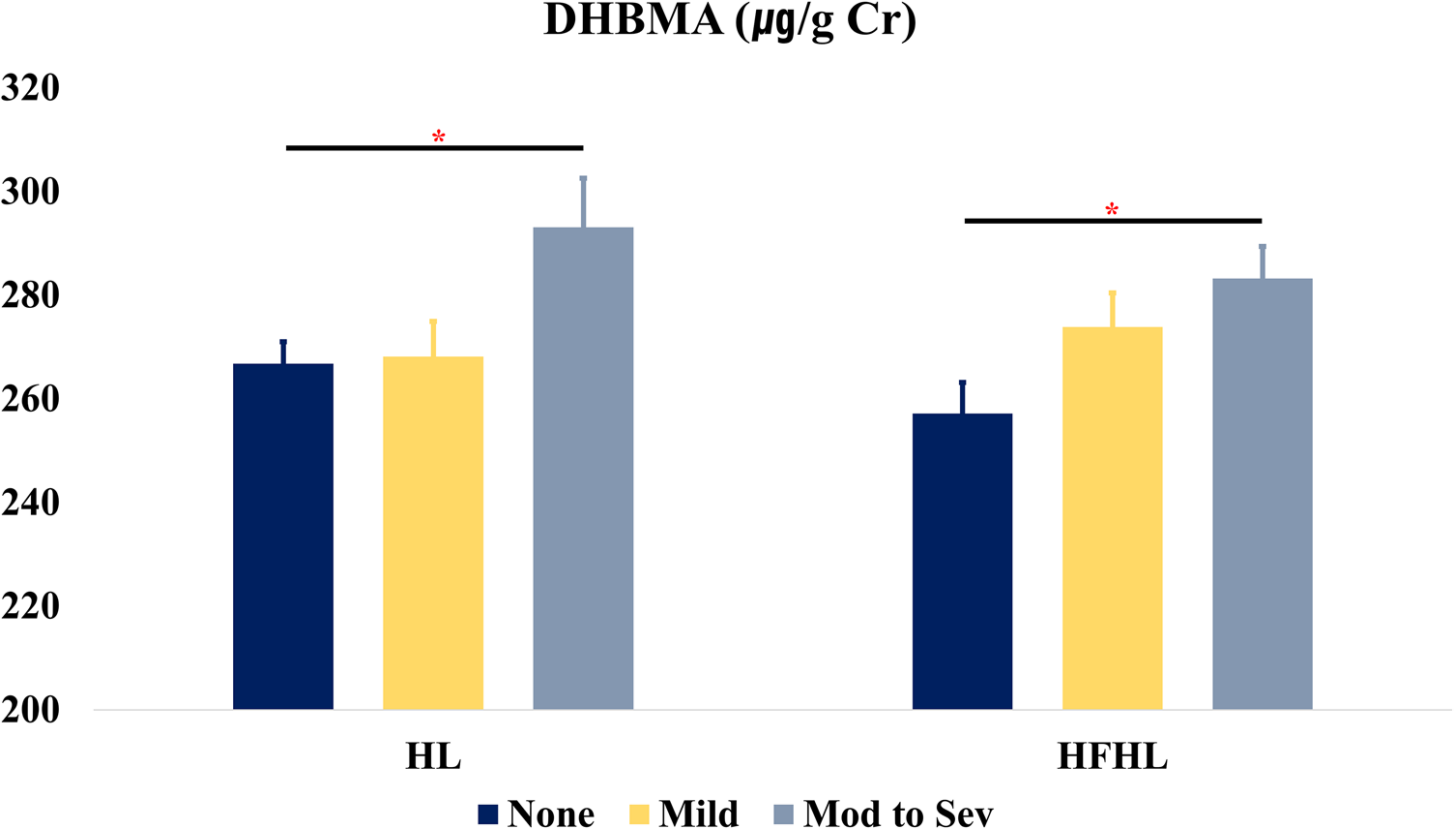
Multivariable-adjusted mean values of DHBMA in the hearing loss and high-frequency hearing loss groups. Cr, creatinine; DHBMA, N-acetyl-S-(3,4-dihydroxybutyl)-L-cysteine; *HL* hearing loss, *HFHL* high-frequency hearing loss, None non-hearing loss group, *mild* mild hearing loss group, *Mod to Sev* moderate to severe hearing loss; *, *P* < 0.05.

After adjusting for age, sex, household income, hypertension, and history of occupational noise exposure, the creatinine-corrected concentrations of DHBMA were associated with HL (*P* = 0.040) and HFHL (*P* = 0.030) (Fig. 2). The average creatinine-corrected concentrations of DHBMA increased with the severity of HL and HFHL (Fig. 2). In the post-hoc analysis, the concentration of DHBMA was significantly higher in the moderate to severe HL group than in the normal hearing group (*P* = 0.048) (Fig. 2). Similarly, the moderate to severe HFHL group showed higher DHBMA concentrations than the normal HF hearing group (*P* = 0.027) (Fig. 2).

### Association between DHBMA and HL or HFHL in the occupational noise exposure group

We selected the occupational noise exposure group to assess the relationship between DHBMA levels and HL or HFHL within this cohort. Within the noise exposure group, there were 97 individuals with normal hearing, 51 with mild HL, and 33 with moderate to severe HL. The creatinine-adjusted DHBMA concentrations varied significantly across these groups (*P* = 0.009). This difference remained significant even after adjusting for age, sex, household income, and hypertension (*P* = 0.024). Furthermore, the group with moderate to severe HL had notably higher creatinine-adjusted DHBMA concentrations than those with normal hearing (*P* = 0.020).

This study included 51, 41, and 89 people with normal HF hearing, mild HFHL, and moderate to severe HFHL, respectively. The noise exposure group exhibited a higher prevalence of moderate to severe HL (49.17%) than the general population (36.75%, *P* = 0.001). However, the creatinine-corrected concentration of DHBMA did not differ significantly among the groups (*P* = 0.184).

### Multivariable linear regression analysis of DHBMA

Since the creatinine-corrected concentration of DHBMA gradually increased according to the severity of HL or HFHL in the multivariable analysis, we evaluated the linear association between the creatinine-corrected concentration of DHBMA and the mean hearing level or mean HF hearing level. After controlling for age, sex, household income, hypertension, and the history of noise exposure, DHBMA showed a significant association with the mean hearing level (β = 0.054, *P* = 0.024) and the mean HF hearing level (β = 0.045, *P* = 0.037) (Table 4).

**Table 4:** Multivariable linear regression analysis between creatinine-corrected DHBMA levels and hearing level or high-frequency hearing level.

Variables	B	SE	β	t	*P*-value
Hearing level					
Age	0.628	0.031	0.556	20.234	** < 0.001**
Sex (female)	− 4.565	0.630	− 0.171	− 7.249	** < 0.001**
Household income (quintile)	− 1.094	0.247	− 0.114	− 4.422	** < 0.001**
Hypertension	0.754	0.713	0.026	1.058	0.290
Occupational noise exposure	1.990	0.817	0.056	2.434	**0.015**
DHBMA (μg/g Cr)	0.007	0.003	0.054	2.257	**0.024**
High-frequency hearing level					
Age	0.970	0.038	0.625	25.249	** < 0.001**
Sex (female)	− 9.378	0.779	− 0.255	− 12.031	** < 0.001**
Household income (quintile)	− 1.246	0.306	− 0.094	− 4.068	** < 0.001**
Hypertension	0.667	0.882	0.017	0.756	0.450
Occupational noise exposure	3.223	1.012	0.066	3.186	**0.001**
DHBMA (μg/g Cr)	0.008	0.004	0.045	2.090	**0.037**

DHBMA, N-acetyl-S-(3,4-dihydroxybutyl)-l-cysteine.

Significants values are in bold.

### Binary logistic regression analysis of the association between hearing loss and DHBMA

We evaluated the effect of DHBMA on HL or HFHL using binary logistic regression analysis after adjusting for age, sex, household income, hypertension, and the history of occupational noise exposure. Based on the results of multivariable analysis, which demonstrated differences between moderate to HL or HFHL compared to the normal hearing group or normal HF hearing group (Fig. 2), moderate to severe degrees of HL or HFHL were classified into HL or HFHL, respectively. After adjusting for other factors, DHBMA showed a significant association with HL (adjusted odds ratio (aOR) = 1.002, *P* = 0.016) and HFHL (aOR = 1.001, *P* = 0.049) (Table 5).

**Table 5:** Binary logistic regression analysis between creatinine-corrected DHBMA levels and hearing loss or high-frequency hearing loss.

Variables	B	SE	Wald	aOR (95% CI)	*P*-value
Hearing loss					
Age	0.099	0.013	60.291	1.104 (1.077–1.132)	** < 0.001**
Sex (female)	− 0.839	0.209	16.195	0.432 (0.287–0.650)	** < 0.001**
Household income (quintile)	− 0.195	0.087	5.068	0.823 (0.694–0.975)	**0.024**
Hypertension	0.038	0.210	0.033	1.039 (0.689–1.568)	0.855
Occupational noise exposure	0.464	0.245	3.577	1.591 (0.983–2.574)	0.059
DHBMA (μg/g Cr)	0.002	0.001	5.506	1.002 (1.0004–1.003)	**0.016**
High-frequency hearing loss					
Age	0.125	0.010	162.355	1.133 (1.112–1.155)	** < 0.001**
Sex (female)	− 1.595	0.179	79.296	0.203 (0.143–0.288)	** < 0.001**
Household income (quintile)	− 0.185	0.065	8.241	0.831 (0.732–0.943)	**0.004**
Hypertension	0.127	0.174	0.531	1.135 (0.807–1.597)	0.466
Occupational noise exposure	0.598	0.209	8.218	1.819 (1.208–2.738)	**0.004**
DHBMA (μg/g Cr)	0.00	0.001	3.881	1.001 (1.000008–1.003)	**0.049**

*aOR* adjusted odds ratio, *CI* conficence interval, DHBMA N-acetyl-S-(3,4-dihydroxybutyl)-l-cysteine.

Significants values are in bold.

### Quantile g-computation analysis of the association between hearing loss and VOCs

**Figure 3:**
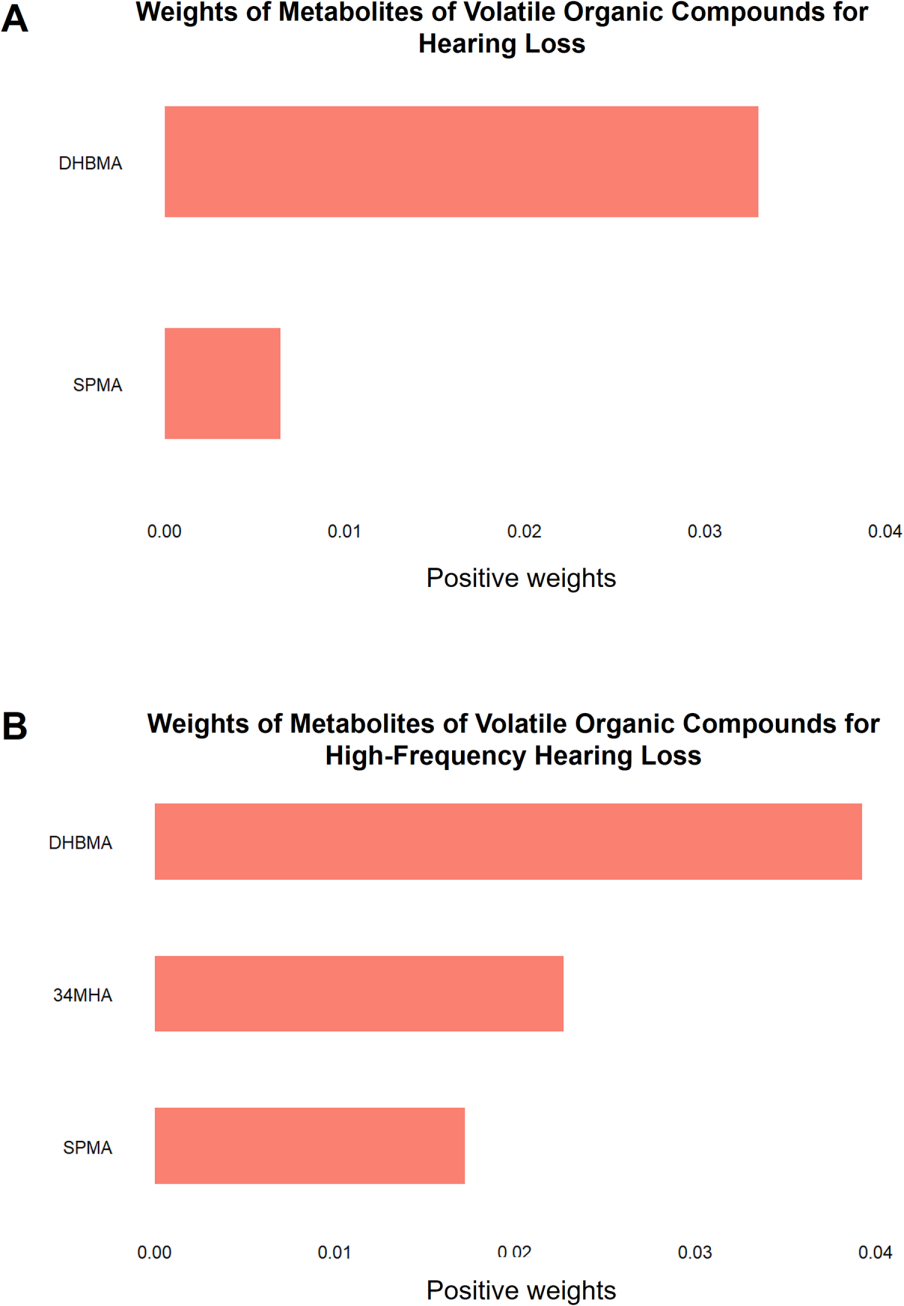
Weights of metabolites of volatile organic compounds for hearing loss (**A**) and high-frequency hearing loss (**B**) in quantile-g computation. *3, 4 MHA*, 3- and 4-methylhippuric acid, *DHBMA* N-acetyl-S-(3,4-dihydroxybutyl)-l-cysteine, *SPMA* N-acetyl-S-(phenyl)-l-cysteine.

Quantile g-computation was performed using VOCs with a p-value less than 0.1 in age- and sex-adjusted analysis. Age, sex, household income, hypertension, and history of occupational noise exposure were included as covariates. Hearing loss (HL) and high-frequency hearing loss (HFHL) were classified using the same classification as in binary logistic regression analysis. Among SPMA and DHBMA, only DHBMA exhibited a significant association with HL after adjusting for other factors (weight = 0.033, *P* < 0.001), while SPMA did not show a significant association with HL (weight = 0.006, *P* = 0.496) (Fig. 3A). Additionally, HFHL also showed a significant association with DHBMA (weight = 0.039, *P* = 0.005) after adjusting for other factors, whereas HFHL did not exhibit a significant association with 3,4 MHA (weight = 0.023, *P* = 0.089) or SPMA (weight = 0.017, *P* = 0.205) (Fig. 3B).

## Discussion

We conducted an analysis to explore the relationships between VOC metabolites and HL, as well as HFHL. Approximately one-third of the analyzed VOC metabolites demonstrated a relationship with HL, and the majority were associated with HFHL. After adjusting for other factors, the creatinine-corrected concentration of DHBMA was found to be associated with both HL and HFHL. However, no other VOC metabolites showed an association with HL or HFHL after controlling for age and sex.

DHBMA is a metabolite derived from 1,3-butadiene^19–21,26,27^, which is a colorless gas and a known carcinogen that has been used in the production of synthetic rubber and plastics^26,27^. Furthermore, 1,3-butadiene can react with DNA, resulting in cancer^20,28,29^. Additionally, it exhibits neurotoxic effects, which may include symptoms such as headaches, blurred vision, nausea, fatigue, paresthesia, and damage to the central nervous system^20,28,29^. It can also induce endothelial dysfunction in vascular structures, which may result in cardiovascular diseases, including hypertension^19–21^. Considering its toxicity to DNA and the nervous system, 1,3-butadiene may also affect the auditory nervous system or hair cells. Since hair cells do not regenerate after being damaged by DNA-binding anticancer drugs such as cisplatin and carboplatin^18,30^, the toxicity of 1,3-butadiene could potentially cause damage to auditory hair cells. Moreover, the neurotoxic effects of 1,3-butadiene may contribute to auditory nerve damage. Additionally, the vascular structure and circulation are critical for hearing^31^, and their impairment can induce HL. Therefore, endothelial dysfunction caused by 1,3-butadiene could play a role in HL or HFHL. Further research is necessary to elucidate the mechanisms by which 1,3-butadiene contributes to hearing impairment and to determine its role in inducing HL.

For workers in industries that manufacture synthetic rubber or plastic, 1,3-butadiene is a significantly hazardous VOC. Therefore, exposure to 1,3-butadiene is strictly regulated to protect workers’ health^32^. In addition, a specialized screening test is available in the Republic of Korea for the early detection of neurological and reproductive abnormalities among workers at high risk. Our findings, which demonstrate a significant association between HL and 1,3-butadiene exposure, suggest that including a hearing test in the current specialized screening could be advantageous. This addition may facilitate the early identification of 1,3-butadiene-associated HL or HFHL and help prevent further auditory damage. Further research is needed to evaluate the causality between 1,3-butadiene exposure and HL or HFHL, which would help determine the benefits of incorporating a hearing test into the specialized screening for workers exposed to 1,3-butadiene.

The general population is also at risk of exposure to 1,3-butadiene through products made from rubber or resins, automobile exhaust, cigarette smoke, and cooking^20^. Much of this non-occupational exposure to 1,3-butadiene takes place indoors or in close proximity to streets. These microenvironments can be managed through proper ventilation or by avoiding areas near streets^20^. Therefore, it is necessary to reduce exposure to 1,3-butadiene, considering its possible ototoxicity. Furthermore, implementing regulations in rubber or resin containing products for 1,3-butadiene can be beneficial in mitigating exposure to potential ototoxic pollutants.

Some previous studies have demonstrated a relationship between VOCs and HFHL in individuals without a history of noise exposure^14,15^. However, these studies did not find a relationship between HFHL and VOCs in individuals with a history of noise exposure. Our study yielded similar results within the noise exposure group with respect to HFHL. These findings may be due to noise-induced HFHL^17,18^, which could potentially mask the effects of 1,3-butadiene. However, we demonstrated that individuals with more severe HL or HFHL exhibited higher concentrations of DHBMA after controlling for occupational noise exposure history. Furthermore, the creatinine-corrected concentration of DHBMA was significantly associated with HL in the noise exposure group. Our findings suggest that managing 1,3-butadiene exposure could be beneficial for HL and HFHL prevention, regardless of noise exposure history.

A limitation of this study was that it did not evaluate the causality between VOCs and hearing thresholds. As we obtained data from a cross-sectional database, we were unable to identify time-dependent changes in hearing thresholds following exposure to higher levels of VOCs. Future studies with a prospective design may be useful in demonstrating the causality between VOCs and hearing thresholds.

Another limitation of our study is the lack of information on other possible covariates for the association between hearing loss and VOCs. We were unable to obtain information about other environmental pollutants such as other organic solvents and heavy metals, as well as occupational factors such as occupation type and the type of industries where participants work from KNHANES. Considering the result of previous study, co-exposure of VOCs and/or heavy metals with noise exposure may aggravate noise-induced hearing loss^13^. Further studies considering other environmental pollutants and occupational factors may provide a more accurate association between hearing loss and 1,3-butadiene exposure.

Additionally, we were unable to evaluate other hearing assessments, such as the bone-conduction hearing test, speech audiometry, otoacoustic emission test, and auditory brainstem response test, as these examinations were not part of the eighth KNHANES. This study can be instrumental in assessing the type of hearing loss and identifying the affected regions of the auditory tract. Further research that includes the results of these audiological tests is essential to elucidate the precise effects and mechanisms underlying the association between 1,3-butadiene exposure and HL or HFHL.

## Conclusion

The creatinine-corrected concentration of DHBMA, a derivative of 1,3-butadiene, was associated with HL, including HFHL. Since 1,3-butadiene may act as an ototoxic substance, early screening for workers exposed to 1,3-butadiene could be beneficial in preventing further HL. Moreover, it is important to reduce exposure to 1,3-butadiene in everyday life due to its potential ototoxic effects.
